# Supplementary figures and images for: Non-gradient and genotype-dependent patterns of RSV gene expression
Source: PLoS One. 2020 Jan 10;15(1):e0227558. doi: 10.1371/journal.pone.0227558 (PMC6953876; doi:10.1371/journal.pone.0227558)

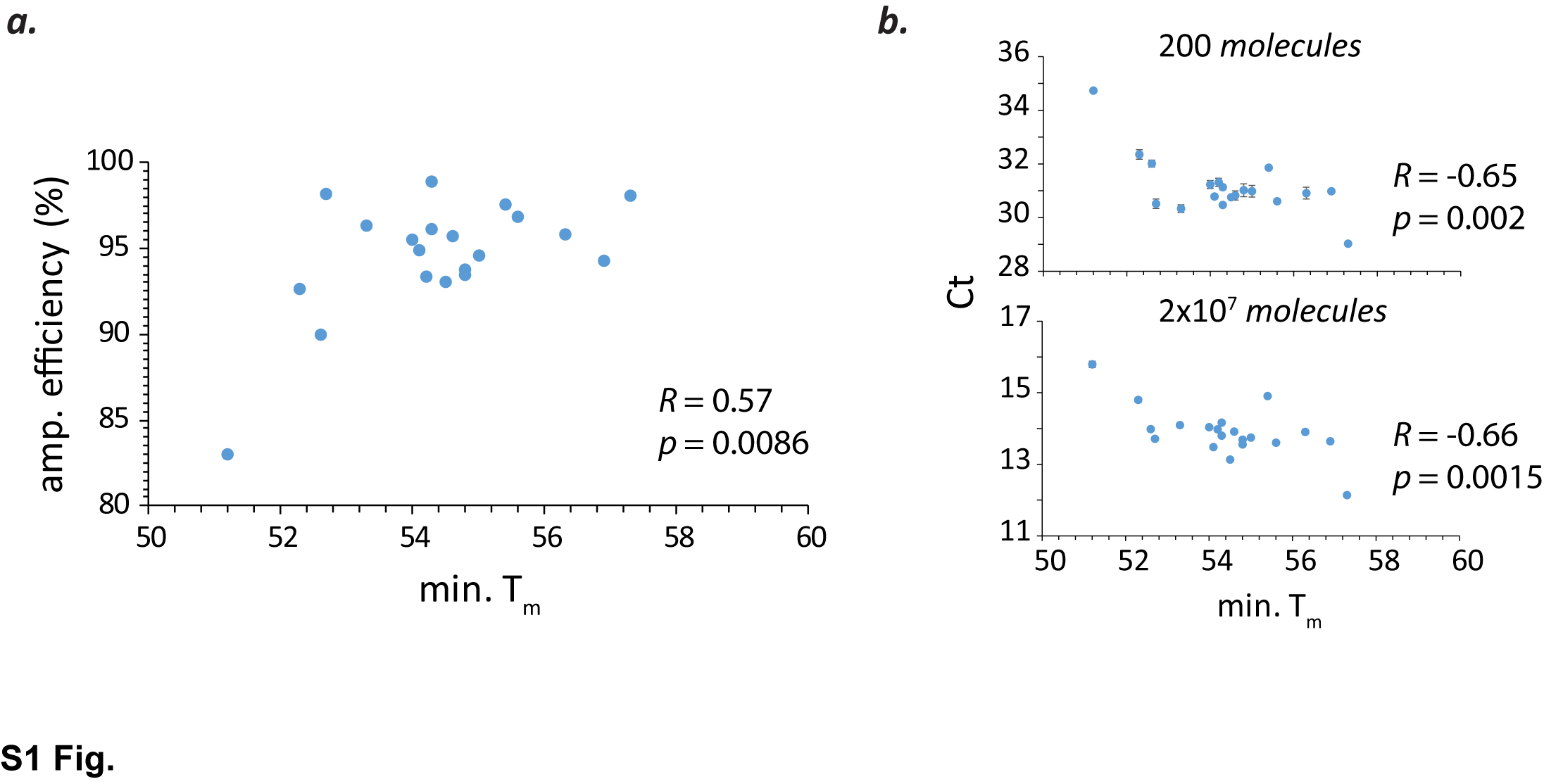

Supplement: S1 Fig — (a) Pearson correlation for amplification efficiencies vs. min. Tm: R = 0.57, p = 0.0086. (b) Pearson correlations for CT values measured at the extremes of target quantity (200 and 2x10^7^ molecules / rxn) vs. min. Tm: R = −0.65, p = 0.002 and R = −0.66, p = 0.0015, respectively. (TIF) [file pone.0227558.s001.tif]

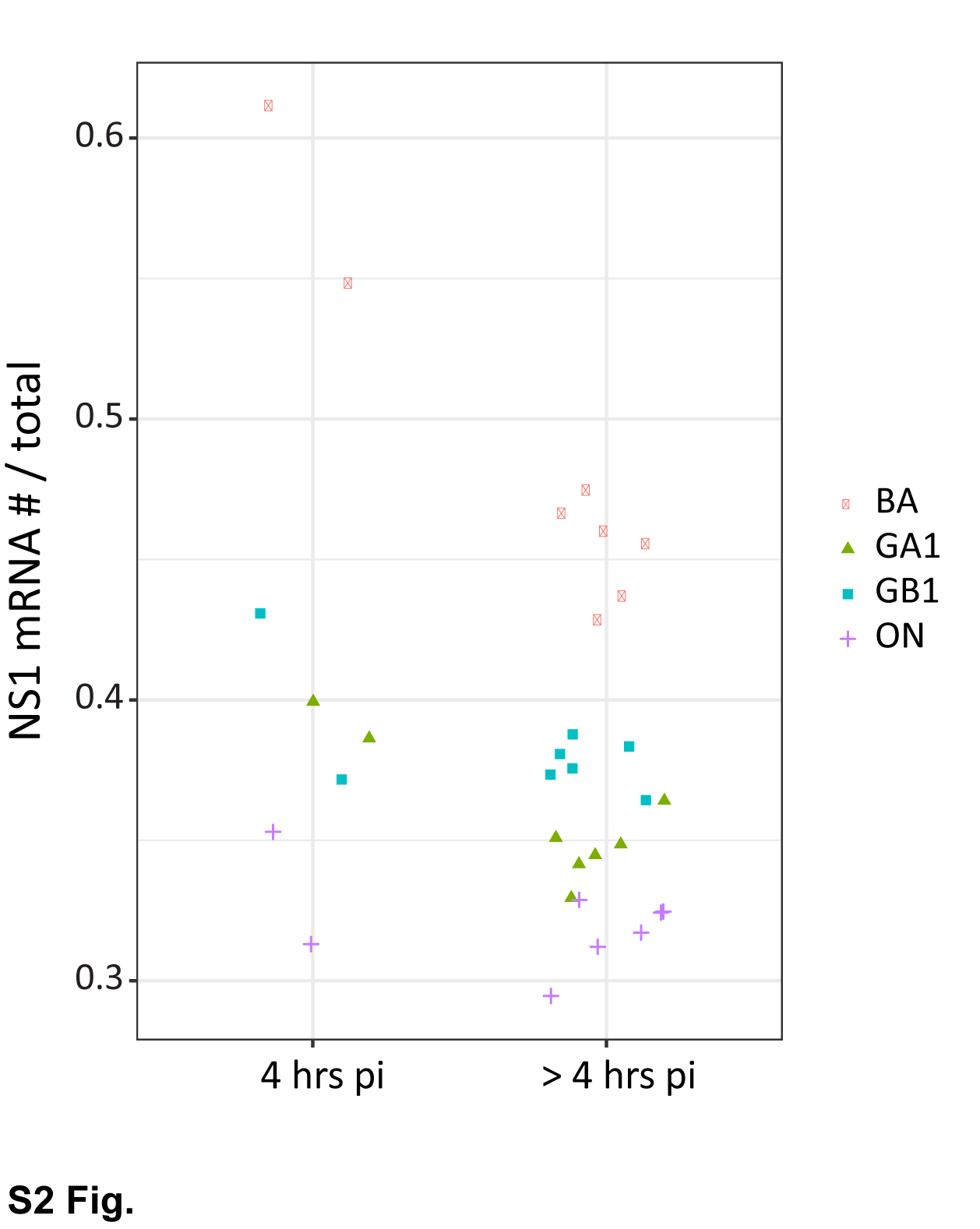

Supplement: S2 Fig — NS1 mRNA # / total vs. time post-infection (4 or > 4 hours). Each plotted point (RSV/A/GA1Tracy [green triangle]; RSV/A/ON/121301043A [purple cross]; RSV/B/GB1/18537 [blue square]; RSV/B/BA/80171 [red box]) represents the mean from duplicate measurements of a single sample. (TIF) [file pone.0227558.s002.tif]
