# Supplementary material for: Non-gradient and genotype-dependent patterns of RSV gene expression
Source: PLoS One. 2020 Jan 10;15(1):e0227558. doi: 10.1371/journal.pone.0227558 (PMC6953876; doi:10.1371/journal.pone.0227558)
Supplement: S1 Table — Primer and probe sequences are shown 5’ to 3’. All reagents were purchased from Integrated DNA Technologies (IDT®). All probes contained the same dye (5’ 6-FAM) and quencher (3’ ZEN). (DOCX) [file pone.0227558.s003.docx]

|  |  | **NS1** | **NS2** |
| --- | --- | --- | --- |
| **GA1** | Fwd | CCAATTTCACAACAATGCCAGTAC | GGCACTTTCCCTATGCCAATA |
|  | Rev | TCATCTAGTAGACCATTAGGTTGAG | GTGTGTTATGATGTCTCTGGTTAGT |
|  | Prb | TGGGAAATGATGGAATTAACACATTGC | TAATCACAGATATGAGACCATTGTCACT |
| **ON** | Fwd | CCAATTTCACAACAATGCCAGTAT | GGCACTTTCCCTATGCCAATA |
|  | Rev | TCATCTATTAGGCCATTAGGTTGAG | ATGTGTTATGATATCTCTGGTTAGA |
|  | Prb | TGGGAAATGATGGAATTAACACACTGC | TGATCACAGACATGAGACCATTATCGCT |
| **GB1** | Fwd | CAGTGAAGTGTGCCCTGACAA | ACCTCATCAAAGGGAAATGGG |
|  | Rev | GACCATTTGATTGAGAGCAGTGTG | GTCTCATGTCTGTGATCATCAATCT |
|  | Prb | CAACTCAATCAATTCCCATATGTATCCTCC | ACTCACCTAATCAGTCAAACCATGAGCACT |
| **BA** | Fwd | CAGTGAAGTGTGCCCTGATAA | ACCTCATCAAAGGGAAATGGG |
|  | Rev | GACCATTTAATTGAGAGCAGTGTG | GTCTCATGTCTGTGATAATCAATCT |
|  | Prb | CAATTCAATCAATTCCCATATGTATCCTCC | ACTCACCTAATCAATCAAACCATGAGCACT |

|  |  | **N** | **G** |
| --- | --- | --- | --- |
| **GA1** | Fwd | GATACACTCAACAAAGATCAACTTCTGTCA | GCAGCAACAATCCAACCTG |
|  | Rev | AGGGGTGTCAATACTATCTCCTGTG | TTTGTGGGCTTGGTGGT |
|  | Prb | TCTAGCAAATACACCATCCAACGGAG | TGGGCCATCTGCAAAAGAATACCC |
| **ON** | Fwd | GATACACTCAACAAAGATCAACTTCTATCA | GCAGCAACAATCCAACCTG |
|  | Rev | AGGAGTGTCAATGCTGTCTCCTGTG | TTTGTGGGCTTGGTGGT |
|  | Prb | TCCAGCAAATATACCATCCAACGGAG | GGGCCATCTGCAAGAGAATACCA |
| **GB1** | Fwd | CTGTGTATAGCTGCACTTGTAATAACC | CACACAAATTCAGCCACAATATC |
|  | Rev | GACATTGTTTGCCCTCCTAATTACTGC | GTGCTTGGCTTGTTGGTCTG |
|  | Prb | AGCAGCAGGAGATAGATCAGGTCTTACA | AGCACAAACCAAAGGCAGAATCAC |
| **BA** | Fwd | GTGTGTATAGCTGCCCTTGTAATAACC | CACACAAACTCAGCCACAATATC |
|  | Rev | GACATTGTTTGCCCTCCTAATTACTGC | GTGCTTGGCTTGTTGTTCTG |
|  | Prb | AGCAGCAGGAGACAGATCAGGTCTTACA | AGCACAAACCAAAGGCAGAACCTC |

|  |  | **F** |
| --- | --- | --- |
| **GA1** | Fwd | GGCATTGCTGTATCTAAGGT |
|  | Rev | GAGGTCTAACACTTTGCTGGTT |
|  | Prb | TCCACAAACAAGGCTGTAGTCAGCT |
| **ON** | Fwd | GGCATTGCTGTATCCAAGGT |
|  | Rev | GAGGTCTAACACCTTGCTGGTT |
|  | Prb | TCCACAAACAAGGCTGTAGTCAGCT |
| **GB1** | Fwd | GCACCACCAACATCAAAGAAG |
|  | Rev | TATCAGCCTGTGGGAAGAAG |
|  | Prb | ACAAGGACTGATAGAGGATGGTATTGTGA |
| **BA** | Fwd | GCACCACCAACATCAAAGAAG |
|  | Rev | TGTCAGCCTGTGGAAAGAAG |
|  | Prb | ACAAGGACTGATAGAGGATGGTACTGTGA |
