# Supplementary material for: Non-gradient and genotype-dependent patterns of RSV gene expression
Source: PLoS One. 2020 Jan 10;15(1):e0227558. doi: 10.1371/journal.pone.0227558 (PMC6953876; doi:10.1371/journal.pone.0227558)
Supplement: Attachment — Submitted filename: Response to Reviewers.docx [file pone.0227558.s005.docx]

December 18, 2019

Dear Dr. Vargas:

Please see the remaining two comments (*in italics*) and our responses (**in bold**) below.

______________________________________________________________________

*Reviewer #1: Piedra et al have adequately addressed most of the stated concerns. Questions about two of the concerns remain, see below.

1) Statistical significance.
As to the example in item 1), the mentioned decrease in NS1 levels after four hours is not clear in Fig. 2. No statistical significance has been added. The norm to determine and communicate the likelyhood that stated differences (whether appearing subtle or not) are real is to calculate p values.*

**We have added a supplementary figure (S2Fig) depicting measured levels of NS1 at 4 hours post-infection (pi) and > 4 hours pi. The noted decrease is statistically significant when analyzed by regression modelling. The manuscript has been modified to incorporate this information (see lines 154-158 and 476-484).**

*2) mRNA ratio variation between cell culture and cotton rats.
* In answering this question, the authors argue that perhaps transcript stabilities cause this difference. However, a general point in the paper is that the non-gradient is not caused by variations in transcript stabilities. These seem to contradict?*

**The data we present indicate that transcript stabilities cannot account for the non-gradient*ness* of the mRNA levels measured, *not* that transcript stabilities cannot at all affect relative mRNA levels. Moreover, *in vitro* transcript stabilities were measured, not *in vivo* stabilities. We therefore do not know what effect (if any) *in vivo* transcript stabilities might have on the difference between RSV mRNA levels in samples from cell culture and cotton rats.**

** This difference between in vitro and in vivo stands out and is interesting and could be important in assessing the value of vitro differences. Therefore the authors should include this finding in the discussion.*

**We agree that the difference between *in vitro* and *in vivo* patterns of RSV transcription is interesting. We have added lines 292-95 to our discussion.**

____________________________________________________________________

Thank you for the chance to respond to the above comments. We submit our revised manuscript and await your decision.

Sincerely,

Dr. Felipe-Andrés Piedra

Dr. Pedro A. Piedra
